# Effects of exercise on kidney function among non-diabetic patients with hypertension and renal disease: randomized controlled trial

**DOI:** 10.1186/1471-2369-13-90

**Published:** 2012-08-28

**Authors:** Franklin C Barcellos, Iná S Santos, Grégore Iven Mielke, Fabrício B del Vecchio, Pedro C Hallal

**Affiliations:** 1 Postgraduate Program in Epidemiology, Federal University of Pelotas, Brazil, Rua Marechal Deodoro, 1160 Pelotas, RS, Brazil; 2 Postgraduate Program in Physical Education, Federal University of Pelotas, Brazil, Rua Luiz de Camões, 625 Pelotas, RS, Brazil

## Abstract

**Background:**

Chronic kidney disease is an important public health threat. Such patients present high morbidity and mortality due to cardiovascular disease, with low quality of life and survival, and also high expenditure resulting from the treatment. Arterial hypertension is both a cause and a complication of kidney disease; also, arterial hypertension is a risk factor for cardiovascular disease among patients with kidney diseases. There is some evidence that exercise interventions may be beneficial to chronic kidney disease patients, but previous studies included only end-stage patients, i.e. those undergoing dialysis. This study aims to evaluate the effect of exercise on kidney function, quality of life and other risk factors for cardiovascular disease among non-diabetic chronic hypertensive kidney disease patients who are not undergoing dialysis.

**Methods:**

The participants will be located through screening hypertensive patients attended within the public healthcare network in Pelotas, a city in south of Brazil. Eligible individuals will be those with glomerular filtration rate between 15 and 59 ml/min x 1.73 m^2^. The randomization will be done in fixed-size blocks of six individuals such that 75 participants will be allocated to each group. At baseline, information on demographic, socioeconomic, behavioral, anthropometric, blood pressure and quality-of-life variables will be collected, and laboratory tests will be performed. The intervention will consist of three weekly physical exercise sessions lasting 60–75 minutes each, with a total duration of 16 weeks. The outcomes will be the kidney function progression rate, quality of life, blood pressure, lipid profile, hemoglobin level, ultrasensitive C-reactive protein level, and ankle-arm index. The patients in both groups (intervention and control) will be reassessed and compared partway through the study (8^th^ week), at the end of the intervention (16^th^ week) and in the 8^th^ week after the end of the intervention.

**Discussion:**

There is still a scarcity of data relating to the effect of physical exercise among the most numerous group of individuals with kidney disease, i.e. patients undergoing conservative treatment. In particular, there is a lack of randomized controlled studies. This study will help fill this gap.

## Background

Chronic kidney disease is an important public health threat [1]. Studies in several countries have estimated that around 12% of the population presents kidney dysfunction (glomerular filtration rate < 60 ml/min x 1.73 m^2^) and that this rate becomes greater with increasing age [2-4]. Patients with chronic kidney disease present high morbidity and mortality due to cardiovascular diseases (DCV) [5], with low quality of life and survival [6].

These patients present diminished kidney function most frequently as a result of diabetes mellitus or systemic arterial hypertension. Systemic arterial hypertension is both a cause and a complication of chronic kidney disease and hypertension is a risk factor for cardiovascular diseases among these patients [7]. Kidney disease patients who are not undergoing dialysis have been studied less often than those receiving dialysis. Strategies are required for keeping these patients’ kidneys functioning and attenuating the risk factors for cardiovascular diseases. Physical inactivity may be an important determinant of morbidity and mortality among these patients [8].

Among the general population, physical activity is associated with lower cardiovascular risk [9]. Individuals of all ages and both sexes benefit from moderate-intensity physical activity, with diminished risk of cardiovascular diseases, maintenance of muscle strength, maintenance of the ability to remain independent, reduction of the risk of falling, fewer symptoms of depression and anxiety and improved quality of life, with increased psychological and physical wellbeing [10]. Physical activity is inversely associated with rates of kidney dysfunction among diabetic patients [11,12]. Despite this evidence, patients with chronic kidney disease tend to be inactive [13]. Moreover, most experimental studies have only included in the intervention group the healthier portion of the patients indicated for kidney replacement therapy, thus making it difficult to extrapolate the results to the whole population undergoing dialysis.

Data on the effect of physical exercise among the most numerous group of individuals with chronic kidney diseases, i.e. patients at less advanced stages of the disease who are still receiving conservative treatment, are still scarce [13]. Furthermore, most studies that have dealt with this influence on chronic renal disease patients were conducted in high-income countries, involving small samples, with short intervention periods, and few were randomized and controlled. There is therefore a need for good-quality data regarding the impact of physical exercise on the prognosis for patients with chronic kidney diseases, and especially in relation to the large group of individuals with kidney diseases in its earliest stages. In addition to the cardiovascular and quality-of-life benefits from interventions, this group also presents the characteristic of potential delay in the progression of nephropathy, which is a benefit that can no longer be expected among the group indicated for kidney replacement therapy. Also, there is an urgent need to reduce the burden of cardiovascular events and the numbers of individuals with indications for dialysis among this population.

### Objectives and main measurements

The aim of the present study is to evaluate the effect of an exercise program on non-diabetic hypertensive patients with chronic kidney disease with regard to disease progression and quality of life. The hypothesis is that patients with chronic kidney disease who perform physical exercises (intervention group) will present less deterioration of kidney function and an increase in the quality-of-life score. Quality of life will be measured by means of the Medical Outcomes Study 36-Item Short Form Health Survey (SF-36), using the Portuguese version translated and validated by Ciconelli [14].

Additionally, the efficacy of the physical exercise will also be assessed by comparing the intervention and control groups regarding the following variables: (a) blood pressure and the need to use antihypertensive medications; (b) serum levels of total cholesterol, HDL cholesterol, triglycerides, albumin, ultrasensitive C-reactive protein, hemoglobin and hematocrit; (c) excretion of proteins and creatinine in urine; (d) ankle-arm index;

## Methods

### Design and setting

A randomized controlled trial will be conducted among patients with chronic kidney disease. The study will be supervised within the Postgraduate Program in Epidemiology in conjunction with the School of Physical Education of the Federal University of Pelotas, which is located in the city of Pelotas, in the south of Brazil. The sample will be composed of non-diabetic individuals presenting systemic arterial hypertension with a glomerular filtration rate of between 15 and 59 ml/min x 1.73 m^2^ (therefore characterized as being in stages 3 or 4 of chronic kidney disease [1]), who are attending primary health care units in the urban zone of the city.

### Recruitment and eligibility

The records of the “HiperDia” project, held by the City Health Department, contain the numbers of individuals with systemic arterial hypertension registered at each primary health care unit in the municipality [15]. After obtaining the names of these patients, their medical files at the primary health care units will be reviewed. Non-diabetic patients with diagnoses of systemic arterial hypertension who in 2009 presented serum creatinine levels greater than or equal to 1.0 mg/dl and glomerular filtration rates (as calculated using the Diet in Renal Disease four variables assessment formula) [16] between 15 and 59 ml/min x 1.73 m^2^ will be identified.

### Exclusion criteria

The following patients will be excluded from the study:

a) Diabetics – we opted to exclude patients with diabetes because they present different prognosis as compared to chronic kidney disease patients without diabetes [17];

b) Individuals with severe physical disabilities (stroke sequelae, lower-limb amputation without a prosthesis, and orthopedic diseases that worsen with exercise);

c) Individuals with a history of acute myocardial infarction over the last six months;

d) Individuals with kidney transplants or who are undergoing dialysis treatment.

### Sample size calculation

Due to the lack of baseline population-based Brazilian data on kidney function, we carried out a pilot study in a subsample of hypertensive patients registered in eight primary health care units in the city of Pelotas in 2010 in order to obtain the estimates needed for the sample size calculation. Through reviewing 3800 medical files, 290 patients with glomerular filtration rates of less than 60 ml/min x 1.73 m^2^ were identified. In this subsample, the glomerular filtration rate of non-diabetic hypertensive patients in stages 3 and 4 of chronic kidney disease was 48.47 ± 9.61 ml/min x 1.73 m^2^. To identify a post-intervention difference greater than or equal to 10% between the intervention and control groups that is significant at the 5% level (two-tailed), with a power of 80% at the end of 16 weeks, it will be necessary to include 63 patients in each group of the study. We decided to expand the sample to 75 patients per group in order to take follow-up losses into account based on previous trials with chronic kidney disease patients. This sample size also allows detecting baseline vs. post-intervention differences of 10% or greater in both groups.

### Random allocation

Random allocation will be performed after the individuals who agreed to participate have been interviewed and have undergone a physical examination. The allocation list will be concealed and patients will be allocated to either the intervention or the control group. In order to balance the groups in terms of numbers of participants, we will use the technique of randomization in fixed-size blocks of six individuals. For each group of six individuals, we will make a draw for the order of entry in each group (intervention or control).

The individuals identified from the “HiperDia” records will be visited at home and invited to participate in the study. After recruitment, a baseline study will be conducted to assess the individuals of both groups before the intervention. The main variables to be collected will be: demographic and socioeconomic data (sex, age, socioeconomic level, schooling level, skin color and marital status); behavioral characteristics (physical activity, smoking and alcohol consumption); anthropometric data (weight, height and waist circumference); clinical data (length of time with diagnosis of hypertension, blood pressure in the upper and lower limbs and anti-hypertensive medications currently used); venous blood and urine sample collection for laboratory analyses (hemogram, lipid profile, blood glucose, serum albumin, ultrasensitive C-reactive protein, proteinuria and creatininuria); and quality of life. The blood and urine samples will be collected at home following an overnight fast (~12 hours) by a nursing technician, and will be sent to the support laboratory, packed in refrigerated boxes at a temperature of approximately 4 °C. Interviewers will be blinded to the interventions or control status of each patient.

**Figure 1:**
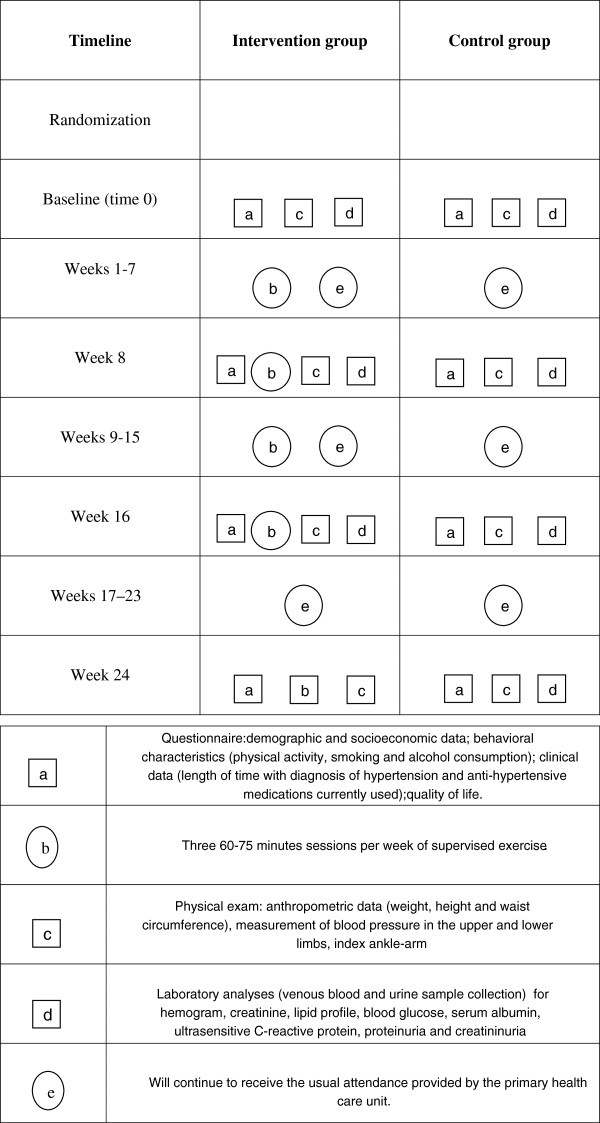
Description of the study protocol.

These patients will be interviewed again at home partway through the study (in the 8^th^ week), just after the end of the intervention (16^th^ week) and in the 8^th^ week after the end of the intervention (Figure 1). At these three visits, a short version of the baseline questionnaire will be applied, and the anthropometric and blood pressure measurements and laboratory tests will be redone. Again, interviewers will be blinded for the intervention or control status of each patient. All the laboratory tests will be performed in a single laboratory, in order to ensure comparability. The questionnaires and the physical examinations will be applied to the participants by previously trained medical students and resident physicians within clinical medical at the School of Medicine of the Catholic University of Pelotas, under supervision by the study coordinator. In all the evaluations, 10 % of the subjects will receive a telephone call from the fieldwork supervisor for quality control purposes.

### Intervention and procedures

The intervention will consist of 16 weeks of physical exercise sessions lasting for an average of 60–75 minutes, at a frequency of three sessions per week. One of the basic principles of exercise training is the progression of intensity, duration or frequency [18]. In our intervention, the progression will be based on two main variables: (a) increases in the effort-rest ratio; (b) reduction in the cool-down time of the sessions, with an increase in the main part of the classes. The workout intensity will be controlled by means of Borg’s Perceived Exertion Scale applied for Resistance Exercise [19].

All patients from the intervention group will be submitted to an acclimation period, in which light and moderate-intensity activities will be prioritized. The intervention will comprise aerobic activities, resistance training and muscle power training. The workout sessions will be composed of an initial section of 5 to 10 minutes of body warm-up and stretching exercises, a main section of circuit or interval training [19-21] with aerobic and resistance exercises lasting for 30 to 35 minutes, and 10–15 minutes of core muscles endurance and flexibility. The final part of the class will be composed of stretching and relaxation exercises lasting approximately 10 minutes.

Resistance training from weeks 1–10 will comprise multi-joint exercises (squat, curved row, bench press, upright row and biceps curl plus shoulder extension), and from weeks 11–16, we will also include low-intensity plyometric training. In terms of muscle power training, we will conduct 2–3 repetitions of 4–6 simple horizontal jumps in the first 14 weeks. In the last two weeks, vertical jumps will also be included [22].

The control group will continue to receive the usual attendance provided by the primary health care unit, in accordance with the guidelines of the Ministry of Health’s primary healthcare manuals for clinical prevention of cardiovascular, cerebrovascular and chronic kidney diseases [23]. The control group patients will answer the same questionnaires and will undergo all the physical examinations and laboratory tests at the baseline, in the 8^th^ and 16^th^ weeks after the start of the intervention and in the 8^th^ week after the end of the intervention, similarly to what will be done with patients from the intervention group.

### Follow-up and adherence to the study

Since the aim of the study is to assess the efficacy of the intervention, strategies for ensuring follow-ups with complete adherence will be used. At the start of the study, the participants will be informed about the importance of the follow-up, and the names, addresses and telephone numbers of two individuals who are close to the participant and who might be able to inform where the participant can be found will be recorded. Over the course of the study, the participants in the intervention group will be contacted by telephone on the day preceding the physical activity sessions. Participants will be offered a snack at the end of each intervention session, transportation vouchers for them to come to the sessions and a pair of training shoes and sports clothes to use at the physical exercise sessions. Participants who do not come to one of the sessions will be sought in their homes to find out why they had been absent. If necessary to ensure adherence, the study will provide door-to-door transportation, free of charge for participants. The intervention will take place between August 2011 and July 2012. The sessions will be administered by physical education professionals, through an agreement with the School of Physical Education of the Federal University of Pelotas, within the premises of the school.

### Primary and secondary outcomes

Progression of kidney disease will be assessed according to glomerular filtration rate measurements, estimated from the four variables formula for Modification of Diet in Renal Disease (MDRD). The glomerular filtration rate will be estimated in the intervention and control groups from serum creatinine measurements obtained at the baseline, in the 8^th^ and 16^th^ weeks after the start of the intervention and in the 8^th^ week after the end of the intervention. Protein excretion in urine will be evaluated through the proteinuria ratio in milligrams per deciliter (mg/dl) and the creatininuria ratio (mg/dl), taking the index to be normal when it is less than 0.3. A secondary outcome is quality of life, which will be measured using the Medical Outcomes Study Short Form 36 item (SF-36). It comprises eight scales, each scored from 0 to 100.

### Measurements

Blood pressure: for measurements of systolic and diastolic blood pressure, the mean from two measurements made five minutes apart, with the patient seated, will be used. In order to maintain precision, an automatic device for blood pressure measurement will be used (Omron HEM-705-CP), and the adult cuff size will be selected (the cuff width should be 40% of the brachial circumference and its length should be 80%). The patients will remain seated, with the arm supported at heart level. They will remain at rest for five minutes before the measurement and should not have had coffee or smoked within the preceding 30 minutes.

Medications: to measure the consumption of medications, the questionnaire standardized by Bertoldi and colleagues [24] will be used. The numbers and names of medications used by the participants will be recorded.

Blood and urine samples: will be collected after the participants have been under fasting conditions for 12 hours. Assays to determine serum levels of total cholesterol, HDL cholesterol, triglycerides, blood glucose, ultrasensitive C-reactive protein, hemoglobin and hematocrit will be performed. Protein and creatinine concentrations will be measured in urine samples. Proteinuria will be assayed using the colorimetric method, with pyrogallol red in a photometric device. Plasma and urinary creatinine will be analyzed by means of kinetics, Jaffe’s method with distilled water and using Roche reagent. Total cholesterol and HDL cholesterol will be measured by means of a colorimetric cholesterol esterase enzymatic method.

Ankle-arm index: this index will be measured with the patient in dorsal decubitus, as the mean of two systolic pressure measurements on the dominant brachial artery (right arm for right-handed individuals and left arm for left-handed individuals), to obtain the proximal pressure; and on the posterior tibial of the ipsilateral leg to obtain the distal pressure [25]. These measurements will be made, respectively, by means of a sphygmomanometer and the Microem DV10 portable vascular Doppler ultrasound equipment (ANVISA/MS registration no. 10301810012). The index is then obtained as the ratio between the distal and proximal pressures.

In addition to these variables, physical activity before and after the intervention will be quantified. Physical activity practice during leisure time and as a means of transport will be evaluated using the long version of the International Physical Activity Questionnaire (IPAQ) [26]. This outcome will assess the sustainability of the intervention in the intervention group, eight weeks after the end of the programmed exercise sessions, in comparison with the level of physical activity in the control group at the same point in time.

### Co-variables

At the baseline, in addition to data on sociodemographic, behavioral and anthropometric characteristics, length of time with the diagnosis of hypertension and laboratory parameters, information on the following factors will be gathered:

1. Medications:

a) Use of antihypertensive agents that block the angiotensin system (angiotensin-converting enzyme inhibitors and angiotensin receptor blockers) that may be associated with diminished progression of kidney disease;

b) Use of non-steroidal anti-inflammatory drugs, which, on the contrary, may increase the progression of chronic kidney disease.

c) Level of physical activity before the start of the study.

### Quality control

The day-to-day progress of the study will be closely monitored by the coordinator, who will be available to resolve any queries that may arise over the course of the fieldwork. The physical exercise sessions of the intervention group will be monitored by a research assistant, who will record the names of those who are present, identify those who are absent and organize home visits within the next 24 hours after the session for the participants who did not attend. According to the reason for the absence, appropriate measures will be taken in order to ensure maximum adherence to the study among the participants.

### Data colleting and analysis

Data will be double-entered into the Epi-Info software in order to make subsequent comparison and thus ensure greater data quality. Automatic data checking will be performed, at the time of data entry, using the check function of Epi-Info, in order to verify range and consistency. To identify and correct inconsistencies of coding, revision and data entry, data cleaning will be performed by obtaining the frequencies of the variables gathered, using the Stata 11.0 software. This software will also be used for the data analysis. A two-tailed significance level of 5 % will be used for all the analyses. The two groups will firstly be compared in terms of the frequencies of the independent variables (demographic, socioeconomic and behavioral factors and time since arterial hypertension was diagnosed), blood pressure levels, glomerular filtration rates and other dependent variables. All analyses will be conducted using intention to treat, i.e., each individual will be analyzed in the group to which he or she was initially allocated, independent of dropouts from the intervention group or any beginning of physical activity among members of the control group. Outcome variables (glomerular filtration rate and quality of life) will be compared in the two groups using *T*-test. We will use confidence intervals for estimating treatment effects.

### Ethical issues

The research ethics committee of the Federal University of Pelotas has approved this study. Likewise, the study has been authorized by the City Health Department and by the coordinators of the health care clinics involved in this study. The results from this study will be communicated to the Health Department of Pelotas and to the Regional Healthcare Coordination Office. Interviewees will be asked to give their written informed consent to their inclusion and will be assured of the right not to answer any part or the whole questionnaire, and their right to withdraw from the study at any time, without any onus on the participant or his/her family.

After the end of the study, if any benefits relating to the disease are found, we will offer the same activity under the same conditions to all participants in the study.

## Competing interests

The authors declare there are no financial competing interests to declare in relation to this manuscript.

## Authors’ contributions

FCB made substantial contributions to conception and design of the study. PH conceived the study, and participated in its design and coordination and helped to draft the manuscript. IS involved in drafting the manuscript and revising it critically for important intellectual content. GM participated in the design of the study. FBV is in charge of the exercise intervention. All authors read and approved the final manuscript.

## Pre-publication history

The pre-publication history for this paper can be accessed here:

http://www.biomedcentral.com/1471-2369/13/90/prepub
